# *Bordetella hinzii* Pneumonia and Bacteremia in a Patient with SARS-CoV-2 Infection

**DOI:** 10.3201/eid2711.211468

**Published:** 2021-11

**Authors:** Michele Maison-Fomotar, Geetha Sivasubramanian

**Affiliations:** University of California, San Francisco, Fresno, California, USA

**Keywords:** bacteria, bacteremia, Bordetella hinzii, Bordetella, cholangitis, coccobacilli, coronavirus disease, COVID-19, endocarditis, pneumonia, respiratory infections, SARS-CoV-2, severe acute respiratory syndrome coronavirus 2, soft tissue infections, urinary tract infections, zoonoses

## Abstract

Patients with severe acute respiratory syndrome coronavirus 2 infection may have bacterial co-infections, including pneumonia and bacteremia. *Bordetella hinzii* infections are rare, may be associated with exposure to poultry, and have been reported mostly among immunocompromised patients. We describe *B. hinzii* pneumonia and bacteremia in a severe acute respiratory syndrome coronavirus 2 patient.

Since the December 2019 beginning of the coronavirus disease (COVID-19) pandemic, caused by severe acute respiratory syndrome coronavirus (SARS-CoV-2), there have been >180 million cases and >3.9 million deaths worldwide (*1*). Severe bacterial and fungal co-infections are a major concern with COVID-19 and increase disease mortality (*2*). 

The genus *Bordetella* comprises >10 known species of small, gram-negative coccobacilli, the most common of which is *Bordetella pertussis* (*3*). *Bordetella hinzii* was first identified as a cause of respiratory infection in poultry and more rarely in rodents (*4*). It was first reported as a human infection in a patient with HIV infection in 1994 as a cause of bacteremia (*5*) and has subsequently been identified as a cause of soft tissue infections, pneumonia, cholangitis, urinary tract infections, bacteremia, and endocarditis, most often in immunocompromised patients (*4*–*15*; Appendix references *16,17*). We report a case of *B. hinzii* pneumonia and bacteremia in a patient with SARS-CoV-2 infection. 

## The Study

A 77-year-old man with medical history notable for uncontrolled type 1 diabetes mellitus and coronary artery disease and who was receiving hemodialysis for end-stage renal disease sought treatment with worsening shortness of breath and 3 days of chest pain. He also reported cough, nausea, fever, and back pain. He lived at a nursing home and had no known poultry or pet exposure. At initial examination, he was afebrile; had a blood pressure of 165/83 mm Hg; heart rate of 92 beats/min, and respiratory rate of 18 breaths/min; was severely hypoxic with oxygen saturation of 50% on room air, requiring a nonrebreather mask; and had decreased breath sounds on chest auscultation. Blood test results (reference ranges) showed hemoglobin, 10 g/dL (12–16 g/dL); leukocytes, 4,300 cells/mm^3^ (4,000–11,000 cells/mm^3^), 78% neutrophils; platelets, 238,000/mm^3^ (140,000–440,000/mm^3^); serum creatinine level, 4.3 mg/dL (0.5–1.1 mg/dL); procalcitonin, 3.3 ng/mL (0.00–0.30 ng/mL); lactate dehydrogenase, 169 U/L (100–230 U/L); C-reactive protein, 213 mg/L (0.0–3.0 mg/L); and ferritin, 2,492 ng/mL (22.0–322.0 ng/mL). A SARS-CoV-2 nasopharyngeal swab sample test was positive by PCR. A computed tomography scan of his chest revealed multiple rib fractures, a large right-side pleural effusion, and right upper-lobe pulmonary infiltrate. 

We started the patient on dexamethasone. We considered remdesivir therapy but did not start it because of his renal disease. We also empirically initiated treatment with piperacillin/tazobactam and levofloxacin for bacterial pneumonia. We performed right-side thoracentesis and drained 725 mL of transudative fluid; fluid culture was negative for growth of bacteria. He was intubated on day 7 after admission because of worsening hypoxemia but subsequently extubated on day 9. On day 13, acute respiratory failure (oxygen saturation ≈70%) and bradycardia (heart rate ≈40 beats/min) developed, and he was hypotensive with agonal breathing. He was emergently reintubated and given atropine, which improved his heart rate. We initiated broad-spectrum antimicrobial treatment with intravenous vancomycin and cefepime.

Blood cultures drawn on day 13 after admission grew gram-negative rods in routine blood, chocolate, and MacConkey agar media. A computed tomography scan of the chest revealed bilateral patchy ground glass opacities, dense consolidations in both lung bases, and a small right pleural effusion (Figure). The patient underwent a bronchoalveolar lavage (BAL) on day 14; the BAL fluid grew >100,000 CFUs of the same gram-negative bacilli, which we had not yet identified, along with 20,000–50,000 CFUs of *Klebsiella pneumoniae*. Gram stain of the BAL fluid showed many leukocytes and few gram-negative rods. We continued treatment with vancomycin and cefepime. On day 17, we extubated then reintubated him the same day because of ongoing hypotension and poor mentation. Because of worsening hemodynamic status, continued poor mentation, and overall poor prognosis, we changed goals of care to comfort measures only, and the patient died soon after. 

**Figure:**
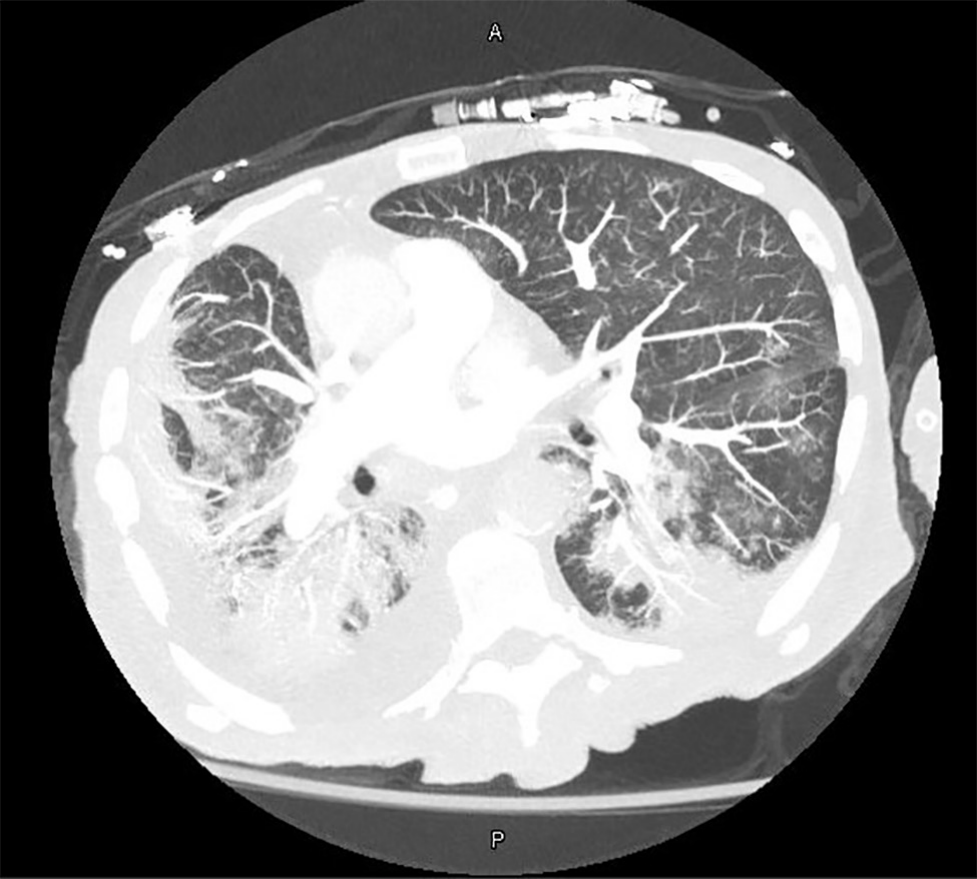
Computed tomography image of the chest showing bilateral dense consolidations and right-sided pleural effusion in 77-year-old man with severe acute respiratory syndrome coronavirus 2 who was later found to be co-infected with *Bordetella hinzii*. A, anterior; P, posterior

On day 18 after the patient’s admission, we identified the gram-negative rod in the blood culture and BAL fluid as *Bordetella hinzii* on the basis of an excellent score (2.43) in matrix-assisted laser desorption/ionization-time of flight mass spectrometry testing. We measured antimicrobial sensitivities by broth microdilution using the Vitek 2 system (bioMérieux; https://www.biomerieux.com) and MIC, interpreting breakpoints using Clinical and Laboratory Standards Institute (https://clsi.org) guidelines. The isolate was sensitive only to meropenem, levofloxacin, amikacin, and gentamicin and showed high MICs of 32 μg/mL to ceftazidime and 64 μg/mL to cefepime (Table 1).

**Table 1:** Antimicrobial susceptibility of *Bordetella hinzii* isolate by broth dilution

Drug	MIC*	Interpretation
Amikacin	8 μg/mL	Sensitive
Aztreonam	≥64 μg/mL	Resistant
Cefepime	≥64 μg/mL	Resistant
Ceftazidime	≥32 μg/mL	Resistant
Ciprofloxacin	≥8 µg/ml	Resistant
Gentamicin	4 μg/mL	Sensitive
Levofloxacin	2 μg/mL	Sensitive
Meropenem	2 μg/mL	Sensitive
Piperacillin/tazobactam	≥128/4 μg/mL	Resistant
Ticarcillin/clavulanic acid	≥256/2 μg/mL	Resistant
Tobramycin	16 μg/mL	Resistant

*MIC breakpoints were interpreted using CLSI guidelines (https://clsi.org).

## Conclusions

*B. hinzii* is a strictly aerobic gram-negative bacillus that was first identified as a cause of respiratory illnesses, mostly rhinotracheitis, in poultry (*3*). Manifestations from reported human cases include skin infection, urinary tract infection, pneumonia, and infective endocarditis, with or without bacteremia (*4*–*15*; Appendix references *16,17*) (Table 2). Human infection with *B. hinzii* is very uncommon; the 18 cases thus far reported suggest that *B. hinzii* behaves like an opportunistic pathogen in humans. Underlying conditions in patients from those cases included HIV, malignancy, liver disease, ulcerative colitis, diabetes, and liver transplantation; 3 of the patients had no underlying medical conditions. There was often known poultry exposure, unlike in this case. It is possible that this pathogen colonizes the respiratory tract then is activated to cause infection later when the host becomes immunocompromised (*7*; Appendix reference *16*). *B. hinzii* was isolated from wild rodents in Southeast Asia, raising the possibility that they might serve as reservoirs that could transmit the pathogen to humans or pets (Appendix reference *18*). Most patients recovered when treated with appropriate antimicrobial drugs, but this infection can lead to death, especially in severely immunocompromised patients (*10*,*13*). 

**Table 2:** Characteristics of previously reported *Bordetella hinzii* infections*

Ref.†	Type of infection	Age, y	Underlying conditions	Animal exposure	Antimicrobial drugs	Patient outcome
(*5*)	Bacteremia	24	HIV/AIDS	None	Ceftazidime	Recovered
(*4*)	Pneumonia	NA	HIV/AIDS	None	NA	NA
(*6*)	Bacteremia and cholangitis	69	None	None	Ticarcillin/sulbactam, ciprofloxacin	Died
(*7*)	Cholangitis	29	Primary sclerosing cholangitis, liver transplant recipient	None	Meropenem	Died
(*8*)	Bacteremia	79	Myelodysplastic syndrome	None	Ceftazidime	Recovered
(*9*)	Bacteremia	36	EBV associated diffuse large cell lymphoma	None	Meropenem	Died
(*10*)	Pneumonia	43	AML, transplant, diabetes bronchiectasis	Poultry	Piperacillin/tazobactam, ciprofloxacin	Recovered
(*10*)	Pneumonia	74	Laryngeal cancer, prostate cancer, diabetes, COPD	None	Piperacillin/tazobactam	Recovered
(*11*)	Urinary tract infection	55	None	Possible poultry	Trimethoprim/ sulfamethoxazole	Recovered
(*11*)	Liver abscess	58	Hypothyroidism, cholecystectomy	None	None	Recovered
(*12*)	Bacteremia and infective endocarditis	79	Aortic valve replacement, diabetes, cirrhosis, colon cancer, kidney disease	None	Meropenem	Recovered
(*13*)	Bacteremia and infective endocarditis	53	Ulcerative colitis	None	Ceftazidime	Recovered
(*14*)	Soft tissue abscess	63	None	None	Sitafloxacin	Recovered
(*15*)	Pancreatic abscess	42	Alcoholic liver disease	None	Tigecycline	Recovered
(*16*)	Urinary tract infection	37	Chronic alcohol use	None	Trimethoprim/sulfamethoxazole	Recovered
(*17*)	Pneumonia	67	Diabetes mellitus	None	Cefmetazole	Recovered

*AML, Acute myeloid leukemia; COPD, chronic obstructive pulmonary disease; EBV, Epstein-Barr virus; NA, not available; ref., reference. †References *16*,*17* in Appendix.

*B. hinzii* is frequently resistant to many antimicrobial drugs, including β-lactams, cephalosporins, and quinolones. Reported isolates have been susceptible to piperacillin/tazobactam, ceftazidime, tigecycline, and meropenem (*4*–*11*). The interpretation of antimicrobial sensitivity testing is not established. Choice of antimicrobial drugs and treatment duration are also not standardized. The cases of bacteremia and endocarditis identified were treated with ceftazidime and ticarcillin/clavulanate. The patient we describe had received only a short course of vancomycin and cefepime before we identified *B. hinzii* in cultures from samples he provided. The isolate of *B. hinzii* identified had a high MIC to cefepime, 64 μg/mL, suggesting inadequate antimicrobial coverage before his death. This high MIC to third- and fourth-generation cephalosporins had been reported in only 1 previous case (*11*). 

The cause of death in this case was likely multifactorial and included respiratory infection with SARS-COV-2 and the hemodynamic compromise that ensued. The role of *Klebsiella* isolated from BAL fluid seems unclear, but this bacterium was found only in very small quantities from the respiratory tract and was treated with appropriate antimicrobial drugs. 

In summary, *B. hinzii* has multiple clinical manifestations and outcomes in both immunocompetent and immunocompromised patients. Reports of patients with *B. hinzii* infections seem to be increasing in recent years, which may be because of the availability of better identification methods, such as matrix-assisted laser desorption/ionization-time of flight mass spectrometry and gene sequencing, as well as an increase in the number of immunocompromised persons who have underlying conditions such as HIV, malignancy, or transplantation or who are taking immunosuppressive agents. Our patient likely had untreated lung *B. hinzii* infection that led to bacteremia. He had uncontrolled diabetes and received dexamethasone as part of his treatment, which may have resulted in dissemination through bacteremia. In addition, SARS-CoV-2 co-infection rendered him more susceptible to infection. Our findings add to the growing knowledge of emerging secondary infectious complications, including from opportunistic pathogens, concurrent with or after SARS-CoV-2 infection. The increasing case reports of invasive *B. hinzii* may indicate its emergence as a pathogen in humans.

Appendix References 16–18 for article *Bordetella hinzii* pneumonia and bacteremia in a patient with SARS-CoV-2 infection.

## References

[1] WHO. COVID-19 dashboard [cited 2021 Jun 28]. https://covid19.who.int

[2] Chen X, Liao B, Cheng L, Peng X, Xu X, Li Y, et al. The microbial coinfection in COVID-19. Appl Microbiol Biotechnol. 2020;104:7777–85. 10.1007/s00253-020-10814-6 32780290 PMC7417782

[3] Mattoo S, Cherry JD. Molecular pathogenesis, epidemiology, and clinical manifestations of respiratory infections due to *Bordetella* pertussis and other Bordetella subspecies. Clin Microbiol Rev. 2005;18:326–82. 10.1128/CMR.18.2.326-382.2005 15831828 PMC1082800

[4] Gadea I, Cuenca-Estrella M, Benito N, Blanco A, Fernández-Guerrero ML, Valero-Guillén PL, et al. *Bordetella hinzii*, a “new” opportunistic pathogen to think about. J Infect. 2000;40:298–9. 10.1053/jinf.2000.0646 10908033

[5] Cookson BT, Vandamme P, Carlson LC, Larson AM, Sheffield JV, Kersters K, et al. Bacteremia caused by a novel *Bordetella* species, “*B. hinzii”.* J Clin Microbiol. 1994;32:2569–71. 10.1128/jcm.32.10.2569-2571.1994 7814500 PMC264104

[6] Kattar MM, Chavez JF, Limaye AP, Rassoulian-Barrett SL, Yarfitz SL, Carlson LC, et al. Application of 16S rRNA gene sequencing to identify *Bordetella hinzii* as the causative agent of fatal septicemia. J Clin Microbiol. 2000;38:789–94. 10.1128/JCM.38.2.789-794.2000 10655386 PMC86205

[7] Arvand M, Feldhues R, Mieth M, Kraus T, Vandamme P. Chronic cholangitis caused by *Bordetella hinzii* in a liver transplant recipient. J Clin Microbiol. 2004;42:2335–7. 10.1128/JCM.42.5.2335-2337.2004 15131227 PMC404647

[8] Fry NK, Duncan J, Edwards MT, Tilley RE, Chitnavis D, Harman R, et al. A UK clinical isolate of *Bordetella hinzii* from a patient with myelodysplastic syndrome. J Med Microbiol. 2007;56:1700–3. 10.1099/jmm.0.47482-0 18033844

[9] Hristov AC, Auwaerter PG, Romagnoli M, Carroll KC. *Bordetella hinzii* septicemia in association with Epstein-Barr virus viremia and an Epstein-Barr virus-associated diffuse large B-cell lymphoma. Diagn Microbiol Infect Dis. 2008;61:484–6. 10.1016/j.diagmicrobio.2008.03.013 18482816

[10] Fabre A, Dupin C, Bénézit F, Goret J, Piau C, Jouneau S, et al. Opportunistic pulmonary *Bordetella hinzii* infection after avian exposure. Emerg Infect Dis. 2015;21:2122–6. 10.3201/eid2112.150400 26584467 PMC4672423

[11] Almuzara M, Barberis C, Traglia GM, Sly G, Procopio A, Vilches V, et al. Isolation of *Bordetella* species from unusual infection sites. JMM Case Rep. 2015;2:e000029. 10.1099/jmmcr.0.000029

[12] González MM, Romano MPC, de Guzmán García Monge MT, Martín BB, García AS. *Bordetella hinzii* endocarditis, a clinical case not previously described. Eur J Case Rep Intern Med. 2019;6:000994. 30931262 10.12890/2019_000994 PMC6432829

[13] Zohourian H, Sorokin AV, Ladna JM, Mushtaq F. *Bordetella hinzii*: an unexpected pathogen in native valve endocarditis. Can J Cardiol. 2019;35:1604.e17–9. 10.1016/j.cjca.2019.08.016 31679629

[14] Negishi T, Matsumoto T, Shinagawa J, Kasuga E, Horiuchi K, Natori T, et al. A case of cervical subcutaneous abscess due to *Bordetella hinzii.* Diagn Microbiol Infect Dis. 2019;95:114865. 10.1016/j.diagmicrobio.2019.07.003 31405631

[15] Kampmeier S, Rennebaum F, Schmidt H, Riegel A, Herrmann M, Schaumburg F. Peripancreatic abscess supported by *Bordetella hinzii.* New Microbes New Infect. 2020;34:100650. 10.1016/j.nmni.2020.100650 32025312 PMC6997295

