# Supplementary material for: Bordetella hinzii Pneumonia and Bacteremia in a Patient with SARS-CoV-2 Infection
Source: Emerg Infect Dis. 2021 Nov;27(11):2904–7. doi: 10.3201/eid2711.211468 (PMC8544983; doi:10.3201/eid2711.211468)
Supplement: Appendix — References 16–18 for article Bordetella hinzii pneumonia and bacteremia in a patient with SARS-CoV-2 infection. [file 21-1468-Techapp-s1.pdf]

Article DOI: https://doi.org/10.3201/eid2711.211468

Bordetella hinzii Pneumonia and Bacteremia in a Patient with SARS-CoV-2

Appendix

Additional References

16. Collercandy N, Petillon C, Abid M, Descours C, Carvalho-Schneider C, Mereghetti L, et al. *Bordetella hinzii*: an unusual pathogen in human urinary tract infection. J Clin Microbiol. 2021;59:e02748–20. PubMed https://doi.org/10.1128/JCM.02748-20

17. Chen D, Wang H, Lu X, Cui Y, Ma X, Lou J, et al. Human pneumonia caused by *Bordetella hinzii*: first case in Asia and literature review. Ann Lab Med. 2021;41:439–42. PubMed https://doi.org/10.3343/alm.2021.41.4.439

18. Jiyipong T, Morand S, Jittapalapong S, Raoult D, Rolain JM. *Bordetella hinzii* in rodents, Southeast Asia. Emerg Infect Dis. 2013;19:502–3. PubMed https://doi.org/10.3201/eid1903.120987
